# Sjogren’s Syndrome Presenting as Hypokalemic Paralysis Secondary to Distal Renal Tubular Acidosis: A Case Report

**DOI:** 10.7759/cureus.72488

**Published:** 2024-10-27

**Authors:** Ashwath Ravisankar, Varshini Thiruvadi, Souwdamini Sethuram, Naveen Prasad Gopalakrishnan Ravikumar

**Affiliations:** 1 Internal Medicine, OSF Saint Francis Medical Center, Peoria, USA; 2 Internal Medicine, Apollo Hospitals, Chennai, IND; 3 Internal Medicine, Thanjavur Medical College, Thanjavur, IND; 4 Nephrology, Nephrology Associates of Yakima, Yakima, USA

**Keywords:** autoimmune disease, distal renal tubular acidosis, recurrent hypokalemia, renal tubular defect, severe hypokalemia-induced paralysis, sjogrens syndrome

## Abstract

Sjogren’s syndrome (SS) is an autoimmune disease affecting multiple exocrine glands such as salivary and lacrimal glands, with focal lymphocytic infiltration. The disease predominantly affects women. The classic presentation is xerostomia and keratoconjunctivitis. In adult populations, tubulointerstitial nephritis, which can manifest as renal tubular acidosis (RTA), is the most prevalent renal manifestation of SS. However, in pediatric populations, RTA only occurs in 7.1-19.2% of cases with renal potassium wasting or hypokalemic paralysis. Here, we describe a case of SS manifesting with hypokalemic paralysis with underlying distal renal tubular acidosis (dRTA).

## Introduction

Sjögren's syndrome (SS) is a systemic autoimmune disorder primarily affecting the salivary and lacrimal glands with focal lymphocytic infiltration of the glandular tissue. The prevalence of SS worldwide is estimated to be around 0.4%. It is predominantly seen in women, in a ratio ranging between 9:1 and 19:1 [1]. The hallmark presentation is xerostomia and keratoconjunctivitis sicca through local exocrine gland involvement. However, it can also manifest with the systemic involvement of a wide variety of organs [2]. Severe hypokalemia, which is characterized by serum potassium less than or equal to 2.5 mmol/L, frequently presents as weakness and muscle pain [3-5]. Severe hypokalemia can have life-threatening consequences, which makes prompt identification and management of its etiology a necessity [3,5,6]. Here, we discuss the case of a middle-aged woman with hypokalemic paralysis due to renal tubular acidosis (RTA), an atypical presentation of SS.

## Case presentation

A 56-year-old Native American woman presented with complaints of generalized muscle weakness for four days, progressing to the point of difficulty getting out of bed. The patient also had a long-standing history of dryness of the eyes and mouth. The patient did not have any associated symptoms such as fever, chills, chest pain, or dysuria. She denied the use of any antibiotics, diuretics, or laxatives in the recent past. She had a past medical history of chronic bilateral knee joint pain that was not worked up. Her family history was positive for rheumatoid arthritis.

On physical examination, the patient had dry skin and was normotensive. Muscle strength was 2/5 in both upper and lower extremities, and deep tendon reflexes were diminished. No sensory deficit, autonomic dysfunction, or cranial nerve deficit were noted.

Laboratory investigations revealed a serum potassium level of 1.2 mmol/L, an anion gap of 9, metabolic acidosis with a pH of 7.2, and bicarbonate of 15 mEq/L. Urine analysis revealed a pH of around 8, a urine potassium-creatinine ratio of around 45 mEq/g, and a positive urine anion gap of 2, which in the setting of hypokalemia suggested urine potassium loss. The diagnosis of distal renal tubular acidosis (dRTA) was strongly considered given the hypokalemia, alkaline urine, and normal anion gap metabolic acidosis. Screening for autoimmune diseases revealed positive ANA, strongly positive anti-Ro (SS-A) 78 u/ml, and negative anti-dsDNA. The possibility of SS as a diagnosis was considered.

The patient was aggressively treated with parenteral potassium, followed by oral potassium citrate. A significant improvement in muscle weakness was observed in two days. Blood pH, serum bicarbonate, and potassium values were also normalized. A rheumatology referral was initiated for possible SS. Schirmer’s testing was abnormal. Given the ocular and oral dryness, abnormal Schirmer’s test, and strongly positive anti-RO (SS-A), a diagnosis of primary SS was made.

 Given the dRTA in the setting of SS, the patient was started on oral prednisone 60 mg daily for four weeks and then tapered to 10 mg daily. The patient followed up in the nephrology clinic after three months, and she is currently asymptomatic. Serum potassium levels have remained normal on oral potassium chloride (KCL) 10 mEq twice daily. Serum bicarbonate levels have remained normal at oral sodium bicarbonate levels of 650 mEq twice daily. The patient is scheduled for another follow-up in three months.

## Discussion

RTA refers to disorders that either affect hydrogen ion excretion, bicarbonate reabsorption, or both. It can be identified by normal anion-gap metabolic acidosis (NAGMA) and a glomerular filtration rate that is largely normal [7,8]. Uncertainty surrounds the exact mechanism by which SS causes RTA [9]. Immunocytochemical examinations of renal biopsies have revealed the complete absence of H-ATPase pumps in the intercalated cells of the collecting tubules, which are largely in charge of proton secretion. This finding offers one possible explanation [10]. High titers of an autoantibody aimed at carbonic anhydrase II are yet another potential mechanism. The generation of hydrogen ions inside cells would be expected to be hindered by this enzyme's inhibition [11]. Renal disease prevalence in SS is estimated to be around 5% [12-14]. The most common type of SS-related renal presentation is chronic tubulointerstitial nephritis, which most frequently manifests clinically as dRTA [13-18]. 

Primary SS can affect the entire nephron, causing both proximal RTA and dRTA [12]. Non-anion-gap metabolic acidosis, alkaline urine, and hypokalemia are the most common clinical manifestations of dRTA. The urine anion gap (Na+K-Cl) can be used as a surrogate to estimate urinary ammonium excretion, which in turn can confirm RTA [19]. In this patient, a positive urine anion gap of 2 indicates a defect in the excretion of excess acid. The presence of NAGMA, hypokalemia, elevated urine pH, and a positive urine anion gap indicate dRTA with predominantly renal loss of potassium. 

Severe, symptomatic hypokalemia should be corrected with KCl [16]. Alkali therapy in the form of 1-2 mmol/kg/day sodium bicarbonate (NaHCO_3_) or potassium bicarbonate (KHCO_3_) can be used to correct acidosis in either the proximal RTA or dRTA [16,17].

SS itself involves several stages in the diagnosis, including the detection of dryness in the mouth and eyes, anti-SSA/Ro and anti-SSB/La antibodies in the serum, and a glandular biopsy [18]. IgG-4-related disease can mimic SS with significant overlap in features. However, patients with IgG-4-related diseases often test negative for anti-SSA/Ro and anti-SSB/La antibodies. The variable presentation of SS may lead to patients being seen by a wide range of healthcare providers, from family physicians to dentists. This necessitates the need for keen clinical acumen for early detection and prompt treatment. 

Despite advancements in our understanding of the disease and early diagnosis, there is no treatment specifically targeting SS. Management involves symptomatic treatment of glandular symptoms, with disease-modifying therapy and immunosuppression reserved for extraglandular manifestations.

## Conclusions

This case report highlights that severe hypokalemic paralysis secondary to dRTA could be a rare presentation of SS. This atypical manifestation emphasizes the importance of considering a systemic autoimmune illness in the differential diagnosis, even when the hallmark signs are not apparent. Early recognition and appropriate treatment may lead to improved patient outcomes.
